# Evolution of Atlantic Meridional Overturning Circulation since the last glaciation: model simulations and relevance to present and future

**DOI:** 10.1098/rsta.2022.0190

**Published:** 2023-12-11

**Authors:** Zhengyu Liu

**Affiliations:** Department of Geography, The Ohio State University,154 North Oval Mall, Columbus OH 43210, USA

**Keywords:** proxy records, water mass, Atlantic Meridional Overturning Circulation instability

## Abstract

The Atlantic Meridional Overturning Circulation (AMOC) and the associated water masses have changed dramatically during the glacial–interglacial cycle. Here, I review some recent progress in the modelling of the AMOC and water masses since the Last Glacial Maximum and discuss the relevance of these past AMOC studies to the present and future AMOC study. Recent studies suggested that Atlantic water masses were constrained by carbon isotopes (δ^13^C) and neodymium isotopes (ε_Nd_), while the strength of the AMOC better was constrained by protactinium/thorium ratio (^231^Pa/^230^Th) and the spatial gradient of calcite oxygen isotopes (δ^18^O*_c_*). In spite of the shallower AMOC at the glacial period, its intensity did not differ substantially from the present because of the cancellation of opposite responses to the rising CO_2_ and the retreating ice sheet.

This article is part of a discussion meeting issue ‘Atlantic overturning: new observations and challenges’.

## 1. Introduction

**Figure 1.**
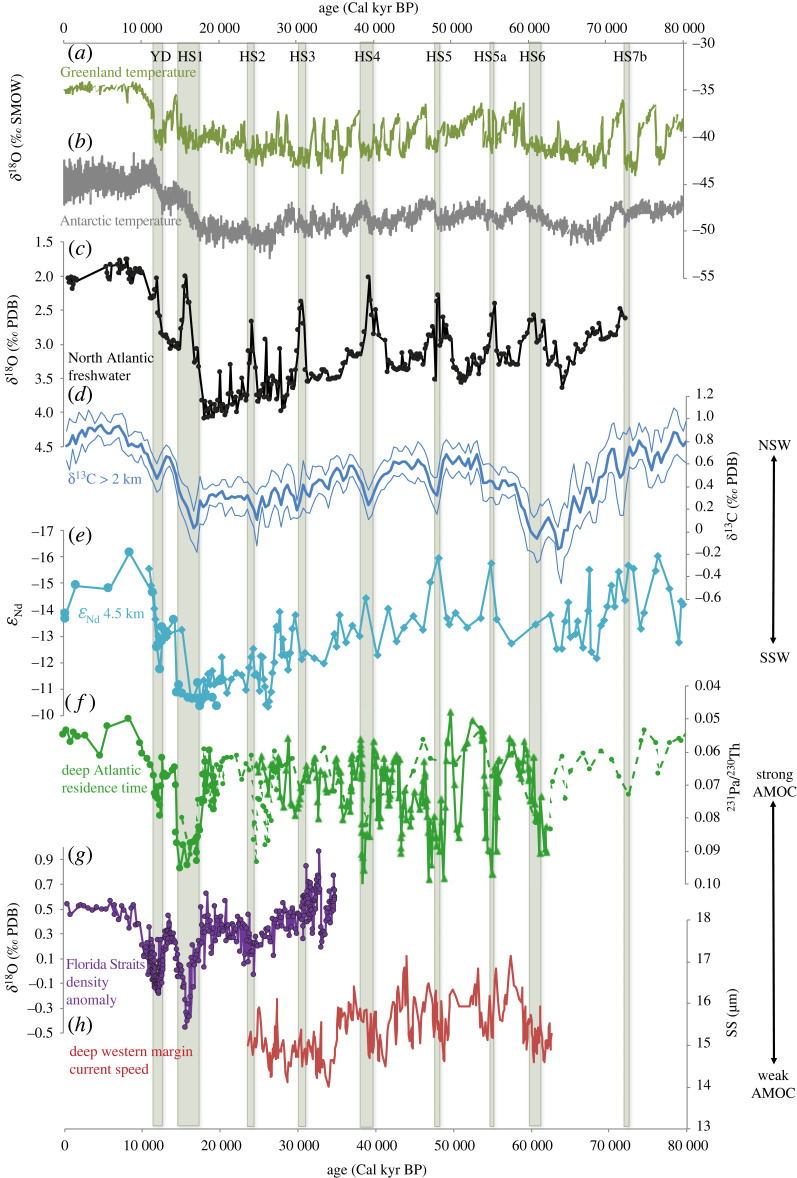
North Atlantic water mass and circulation proxy records over the Heinrich events, along with temperature proxies over Greenland and Antarctica. (*a*) Greenland ice core δ^18^O, (*b*) Antarctica δ^18^O, (*c*) the oxygen isotope ratio in the planktonic foraminifer Neogloboquadrina pachyderma from the western North Atlantic; low values reflect the presence of glacial meltwater. (*d*) Mean and 2-sigma standard-error δ^13^C values (thick and thin lines, respectively) for the deep North Atlantic (greater than 2 km). (*e*) The deep (4.55 km) Nd isotope ratio (*ε*_Nd_) at the Bermuda Rise. (*f*) The ^231^Pa/^230^Th ratio, which can reflect changes in deepwater residence time, from the deep North Atlantic at the Bermuda Rise. (*g*) The ice-volume-corrected oxygen isotope ratio of benthic foraminifera on the Florida Margin, which can reflect changes in the density structure in the Florida Straits and the strength of the upper branch of the Atlantic meridional overturning circulation (AMOC). (*h*) The mean sortable silt grain size along the western boundary of the North Atlantic at the depth of today's North Atlantic Deep Water, which can indicate the current speed on the deep western margin; a large mean size indicates a vigorous flow. See Lynch *et al*. [3] fig. 4 for more details. (Curtesy of J. Lynch-Stieglitz).

Paleoclimate proxies suggest that the Atlantic Meridional Overturning Circulation (AMOC) and the associated water masses have experienced dramatical changes in the past with magnitudes much beyond those in the instrument period. Furthermore, these AMOC changes were accompanied by climate changes and abrupt events over the globe [1,2]. Figure 1 shows multiple proxies relevant to the AMOC, its forcing and its climate impact, for the last 80 000 years [3]. The meltwater flux to the North Atlantic is characterized by strong millennial changes superimposed on the last glacial–deglacial cycle, as seen in the oxygen isotope ratio δ^18^O $\left({∼}^{18}\text{O}{\text{/}}^{\text{16}}\text{O}\right)$ over surface North Atlantic (figure 1*c*). A more depleted (lower) surface water δ^18^O indicates a greater meltwater flux, due to the contribution of more meltwater from ice sheets that have very depleted δ^18^O relative to the average ocean water (−30‰ versus 0‰). Similarly large variability is also evident in two proxies of water masses (δ^13^C, *ε*_Nd_ in figure 1*d,e*) and three proxies of circulation (^231^Pa/^230^Th, δ^18^O, grain size, figure 1*f–h*). The carbon isotope composition ^13^ C/^12^C, expressed as δ^13^C, and the neodymium isotopic composition ^143^Nd/^144^Nd, expressed as *ε*_Nd_, from the shells of benthic foraminifera are two proxies for deep water masses, because their value in the deep water is determined by the competition between the high δ^13^C/low *ε*_Nd_ North Atlantic Deep Water (NADW) source water and the low δ^13^C/high *ε*_Nd_ of the Antarctic Bottom Water (AABW) source water [3–5]. The different behaviour of uranium decay-series nuclides of protactinium and thorium, expressed as ^231^Pa/^230^Th, from sediments in the North Atlantic is considered a proxy of the rate of deep Atlantic circulation, because ^231^Pa has a longer residence time than ^230^Th (111 years versus 26 years) such that the sediment ^231^Pa/^230^Th decreases with increased export of Atlantic deep water [6]. The variability of benthic calcite δ^18^O*_c_* is related to sea water temperature and salinity, and is therefore related to density anomaly and, in turn, ocean current strength through the thermal wind relation [7]. The grain size in the deep western boundary current region (DWBC) is a proxy of DWBC strength, because a coarser grain size is likely to be caused by a stronger current. Finally, large variability is also found accompanying deep ocean changes in the proxy for surface air temperature in Greenland and Antarctica ice cores as indicated in the stable water isotope composition in precipitation δ^18^O, which represents annual temperature via the ‘temperature effect’ associated with Rayleigh distillation [8] (figure 1*a,b*). Overall, these large changes of the AMOC and the associated climate provide a unique opportunity for understanding large AMOC changes and its global climate impact not only for the past, but also for the present and future.

Model-data comparison is important for validating climate models as well as for understanding past AMOC changes. Much progress has been made in modelling and understanding past AMOC changes in the last four decades. Now, state-of-the-art earth system models can be compared with proxy records directly on multiple geo-tracers for the evolution over orbital time scales. This paper is an attempt to review some recent progress on the modelling and model-data comparison of past AMOC changes during the last deglaciation, from the Last Glacial Maximum (LGM, 21 000 years ago) to the preindustrial period (denoted here as the present day or PD). Global climate, the AMOC and water masses have all experienced dramatic changes during this period (the last 21 000 years in figure 1). Temperatures in Greenland and Antarctica warmed up significantly from the LGM to the early Holocene (about 11 000 years ago; figure 1*a,b*), with a 6°C warming in global mean surface air temperature [9] and a 3°C in global mean sea surface temperature (SST) [10], mainly in response to the rising atmospheric CO_2_ and the retreating of ice sheets. Meanwhile, two millennial cooling events of the Heinrich Stadial 1 (HS1, 19 000–14 500 years ago) and Younger Dryas (YD, 12 800–11 800 years ago) bracketed the Bølling–Allerød warming event (BA, 14 500–12 800 years ago) in Greenland (figure 1*a*) and over the Northern Hemisphere [10]. Relatively, temperatures stayed relatively stable in the Holocene (after 11 000 years ago; figure 1*a,b*). The proxies of the AMOC and deepwater masses also experienced great changes during the last deglaciation (figure 1*c–h*). The last deglaciation period provides the best target period for studying the AMOC changes. First, the range of the variability of the AMOC and climate in the last 21 000 years is comparable with that during the entire last glacial–interglacial cycle (figure 1). Second, this period has much more comprehensive proxy records for the climate and ocean than any earlier period (e.g. [3,11]). Finally, the length of this period is becoming practical to be simulated in state-of-the-art earth system models in the synchronously coupled mode, which is critical for the understanding of deep ocean evolution. In this paper, I will review some recent progress in the modelling and understanding of the AMOC changes in the last 21 000 years, as well as a personal perspective on the relevance to the AMOC changes in the future. I will confine my discussion only to the ocean circulation evolution processes, instead of their impact on climate and carbon cycle, which has been discussed extensively (e.g. [2,12–14]).I will not review AMOC instability either, which has been reviewed extensively previously [15–18].

**Figure 2.**
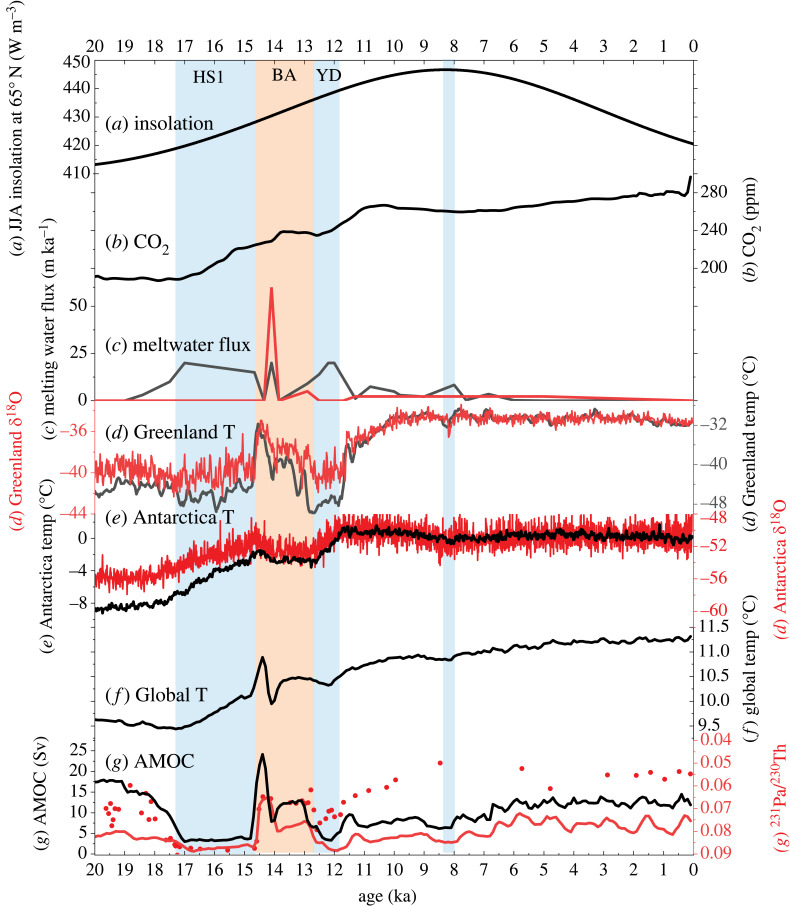
Climate forcing and climate evolution in TRACE. (*a*) Summer insolation at 65°N, (*b*) Atmospheric CO_2_, (*c*) meltwater flux into the North Atlantic (black) and near Antarctica (grey), (*d*) Greenland temperature (black) and ice core δ^18^O (red), (*e*) Antarctica temperature (black) and ice core δ^18^O (red), (*f*) global surface temperature, (*g*) AMOC (black) and ^231^Pa/^230^Th in the model (red) and observation (red markers) [6].

I organized the review mainly around two transient simulations of the AMOC in the last 21 000 years in a comprehensive earth system model, while discussing other relevant works. These two transient simulations, which have been shown in reasonable agreement with many oceanic proxy records, provide a consistent framework for understanding the AMOC evolution. The first is the *Tra*nsient simulation of the *C*limate *E*volution (TRACE, [19]) in a coupled global climate model (CGCM) and the second is the accompanying ocean-alone model simulation called the Carbon isotope enabled TRACE (CTRACE, [20]). TRACE is forced by realistic external forcing of continental ice sheet, greenhouse gases, orbital forcing and melting water fluxes in the fully coupled Community Climate System Model version 3 (CCSM3, figure 2, [19]). CTRACE is the ocean-alone model simulation forced by the surface climate forcing derived from TRACE. CTRACE uses the ocean component model of CESM1, the Parallel Ocean Program version 2 (POP2), which is further coupled with a marine biogeochemical model that incorporates major paleo-geotracers, such as carbon isotopes ${}_{}^{13}\text{C}$, ${}_{}^{14}\text{C}$ [21], neodymium isotopes ^143^Nd and ^144^Nd [22], stable water isotopes ${}_{}^{16}\text{O, }{}_{}^{\text{18}}\text{O}$ [23] and protactinium and thorium ^231^Pa and ^230^Th [24]. Both ocean models have a coarse resolution of a nominal 3° horizontal resolution. The incorporation of geo-tracers in a transient simulation forced by realistically evolving climate forcing facilitates a direct model-data comparison, while the simulation of other idealized tracers further improves the understanding of the physical implications of these geo-tracer proxies on circulation and water masses. In TRACE, AMOC transport evolution is characterized by two large millennial collapses during HS1 and YD (figure 2*g*) forced by meltwater pulses (figure 2*c*), and two recoveries in BA and Early Holocene. The AMOC transport evolution largely follows the ^231^Pa/^230^Th proxy for the AMOC, in the observation and in the CTRACE model (figure 2*g*). This model-data agreement, along with some other agreements that will be discussed later, give some confidence on the relevance of these model simulations to the real world.

The review is arranged as follows. In §2, I will review the deglacial change of Atlantic water masses and the associated the geometry (depth) of the AMOC by first comparing proxy data directly on multiple geo-tracers related to water masses, and then discuss the mechanism of water mass evolution. In §3, I will review the deglacial change of the AMOC strength, also by first comparing proxy with models on AMOC strength-related proxies and then examine the mechanism for the deglacial AMOC transport. A summary and perspective are given in §4.

## 2. Atlantic water masses since the last glaciation

### (a) Proxy constraint on water masses

**Figure 3.**
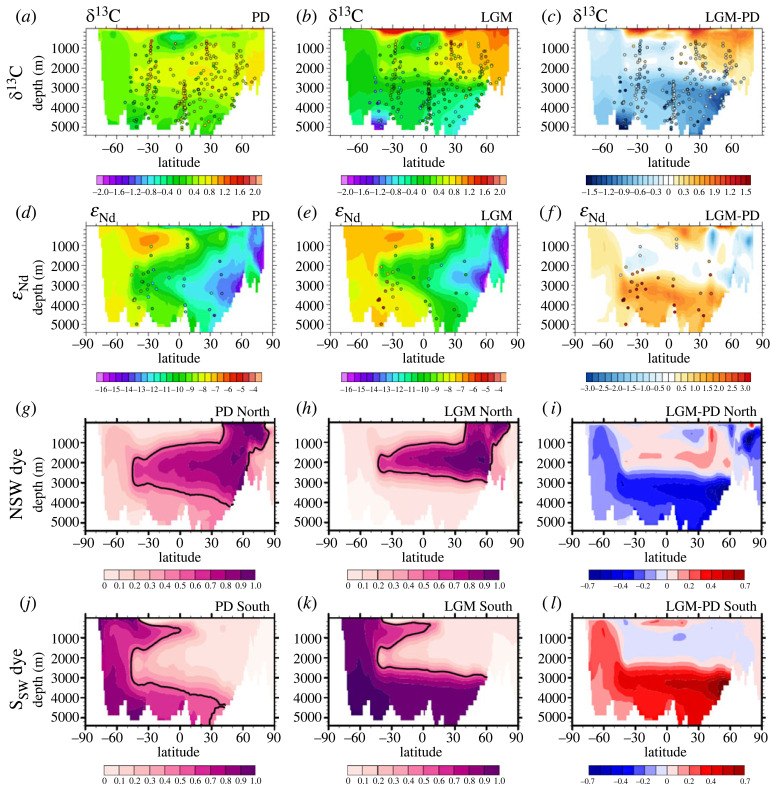
Modelled Atlantic tracer distributions during PD (first column), LGM (second column) and the differences between the LGM and the PD (third column). (*a–c*) Atlantic zonal mean δ^13^C. (*d–f*) Atlantic zonal mean ε_ND_. Observations (LGM and Holocene) are overlaid using the same colour map. Percentage of water originating from (*g–i*) the North Atlantic, (*j–l*) the Southern Ocean. The thick black contours on the LGM and the PD water percentage represent the value of 50%. See a more detailed explanation in Gu *et al*. [27] (adapted from Gu *et al*. [27]).

Multiple proxies suggest that Atlantic water masses have very likely experienced dramatic changes from the LGM to PD [25,26]. One early proxy that has been used to infer water masses is carbon isotope composition δ^13^C $({∼}^{13}\text{C}{\text{/}}^{\text{12}}\text{C)}.$ Primary producers in the surface ocean take up both nutrients and carbon, preferably taking up light isotope such that surface water has low nutrients and high δ^13^C, which is then ventilated into the NADW. By contrast, the AABW is characterized by high nutrients and low δ^13^C, reflecting its long ventilation time away from the surface. At present, the observed δ^13^C is characterized by a high δ^13^C tongue of the NADW overlaying a low δ^13^C tongue associated with the AABW (figure 3*a*, circles). At the LGM, however, the high δ^13^C tongue of NADW shallowed by an approximately 600 m, such that the deep ocean below 2000 m was dominated by the low δ^13^C of the AABW (figure 3*b,c*, circles). This shallowing of high δ^13^C tongue is simulated well in CTRACE (figure 3*a–c*, shading, [27]), with a spatial correlation with paleo observations [28,29] of approximately 0.8 at the LGM, comparable with a previous simulation in the UVic model [30]. This shallowing of high δ^13^C tongue has been suggested as robust evidence of the shallowing of NADW earlier in observations [4,26] and later in many studies, including an inverse modelling [31].

**Figure 4.**
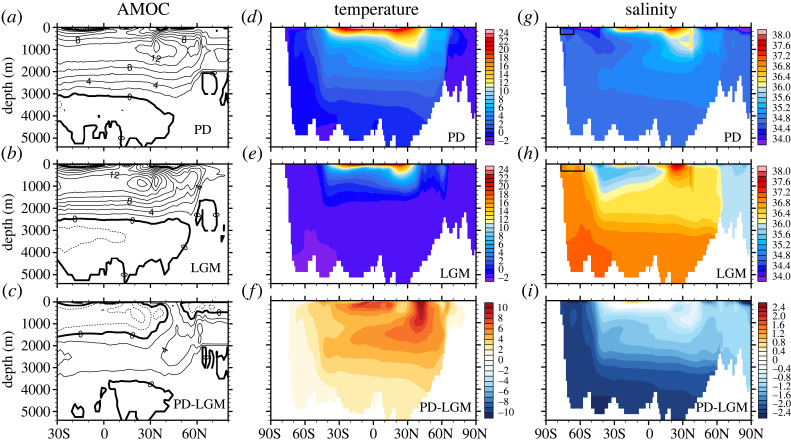
TRACE AMOC overturning streamfunction for (*a*) PD, (*b*) LGM and (*c*) the difference LGM-PD, (*d–f*) and (*g–i*), the same as (*a–c*) but for Atlantic zonal mean temperature and salinity, respectively. Zonal mean sea ice 50% coverage is also marked for PD and LGM in (*g*) and (*h*), respectively. Curtesy of S. Gu (adapted from Gu *et al*. [27]).

**Figure 5.**
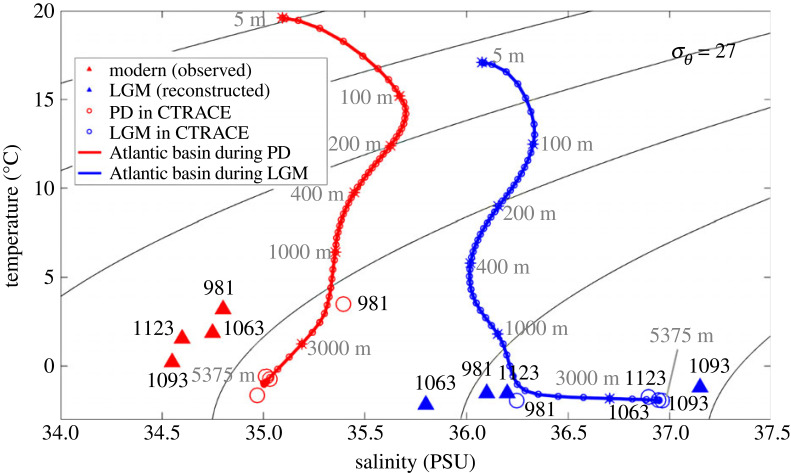
TRACE Atlantic basin mean T-S diagram for PD (red) and LGM (blue). Solid triangles represent the temperature and salinity reconstructions [32] at four deep sites at PD (red) and LGM (blue), while circles represent the model temperature and salinity at the same four sites for PD (red) and LGM (blue). Curtesy of J. Du.

This shallowing of glacial NADW was confirmed in CTRACE clearly in the change of idealized dye tracers between the LGM and PD, with the dye tracer for NADW (figure 3*g–i*) replaced by the dye tracer for AABW at depth (figure 3*j–l*). This shallowing was also seen consistently in the change of AMOC overturning streamfunction from the PD to the LGM in CTRACE, which shallowed by about 600 m (figure 4*a,b*), comparable with the shallow δ^13^C tongue. As such, glacial deep ocean water mass was dominated by the greatly expanded AABW. This change of abyssal water mass in CTRACE was also accompanied by a dramatic charge in the temperature-salinity structure. In contrast to the PD, the AABW at the LGM was so saline that it was even more saline than the NADW, as seen in the T-S diagram of the vertical profiles of ocean temperature and salinity averaged over the Atlantic basin (figure 5). At the PD, deep Atlantic is characterized by two-end members, with the warm/saline NADW kink over the cold/fresh AABW (largely fresher than approx. 35.5 psu), beneath a shallow salinity minimum of Antarctic Intermediate Water (AAIW). At the LGM, however, the AABW was not only the coldest, but also the most saline water mass (up to 37 psu, including approx. 1 psu global increase of salinity associated with the lowering of sea level of 120 m). As such, the T-S curve extended monotonically with depth without the salient kink of warm/saline NADW at all. This T-S change appeared consistent with the glacial salinity pore fluid reconstruction of abyssal temperature and salinity (figure 5, markers, [26,32]), although the abyssal salinity data available (four sites so far) are too sparse to constrain the salinity distribution of the deep Atlantic water masses with high confidence.

The shallowing NADW at the LGM was also consistent with a more recent proxy of water mass: the neodymium composition ε_Nd_ (approx. ^143^Nd/^144^Nd, figure 3*d–f*). The ε_Nd_ is a quasi-conservative tracer without bio-perturbation and is generated mainly by the continental slope (above 3000 m), with the highest value in the North Pacific and the lowest value in the North Atlantic [5]. As such, in PD observations, the NADW shows a low ε_Nd_ tongue while the AABW shows a high ε_Nd_ tongue (figure 3*d*, circles). At the LGM, the low ε_Nd_ tongue associated with the NADW was shifted upward by the high ε_Nd_ water associated with the AABW in observations [33] (figure 3*e*,*f*, circles). This shallowing of low ε_Nd_ tongue has been simulated in two models [27,34], including CTRACE (figure 3*d–f*, shading). Overall, the good model-data agreement on δ^13^C and ε_Nd_ in CTRACE supports the two tracers as useful proxies for water mass geometry, qualitatively [27].

More quantitatively, however, both δ^13^C and ε_Nd_ have deficiencies in representing the geometry of the NADW and AABW. The δ^13^C is affected by biological activities substantially. The biological impact, nevertheless, can be filtered by removing nutrient content (for example, estimated by PO_4_), defined as δ^13^C_AS_ [35] such that δ^13^C_AS_ can be used to track water mass distribution more accurately [36]. In CTRACE, water mass composition estimated from δ^13^C_AS_ tracked that from dye tracers excellently, once the end member values were given for both the NADW and AABW [27]. The NADW% estimated from δ^13^C_AS_ correlated with that derived from the dye tracers at approximately 0.95 for both the PD and LGM, confirming δ^13^C_AS_ as a high-quality tracer for water masses. In comparison, the NADW% derived from ε_Nd_ correlates with that from the dye tracers at only approximately 0.5, with an overestimation of the NADW% in ε_Nd_ at the LGM [27]. This overestimation of NADW at depth may have led to the wrongful interpretation in observations that there was significant abyssal NADW in the North Atlantic [33]. This overestimation in ε_Nd_ is caused by several factors. One factor is that ε_Nd_ has the major unradiogenic source deep from the continental slope of the North Atlantic, unlike the true dye and δ^13^C_AS_ which has the source from the surface [22,34].

**Figure 6.**
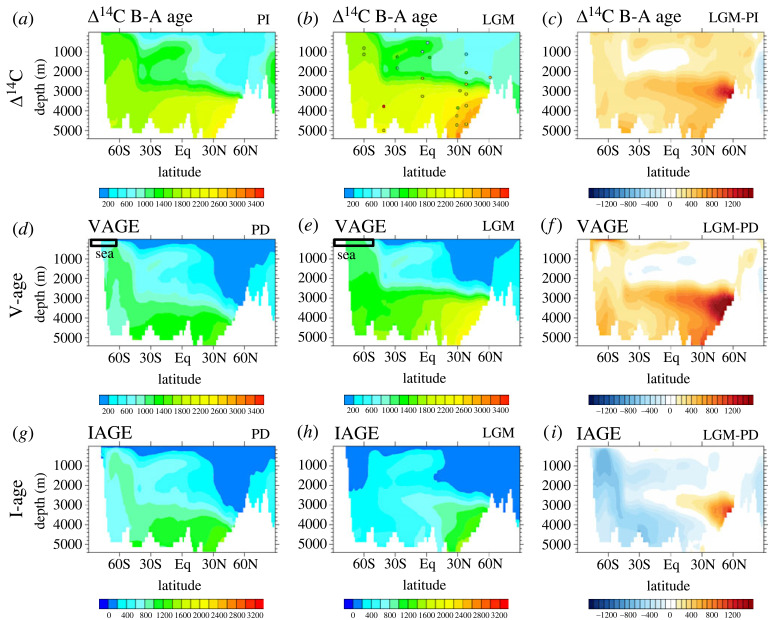
The Atlantic zonal mean water ages during PD, LGM and the differences between the LGM and the PD: radiocarbon ventilation age (*a–c*), idealized ventilation age (*d–f*) and ideal age (*g–i*). The observational LGM radiocarbon age (circles in (*b*)) is from the compilation in Menviel *et al*. [37]. Zonal mean sea ice 50% coverage is also marked for PD and LGM in (*d*) and (*e*), respectively. See Gu *et al*. [27] for more details (adapted from [27]).

The change of water mass depth can also be detected in the water age tracer Δ^14^C. As shown in figure 6*a–c*, the CTRACE ocean had an older Δ^14^C age at the LGM than the PD, in terms of the difference between benthic Δ^14^C and atmospheric Δ^14^C, or B-A age, in deep water (figure 6*a–c*). The maximum difference occurred at 3 km depth, where the shallower NADW was replaced by a, presumably, more sluggish AABW, consistent with observations. CTRACE, nevertheless, failed to reproduce the very old deep water in the South Atlantic as indicated in a single proxy data (red circle at 3700 m, 45° S, figure 6*b*), which corresponds to the deep ‘bulge’ of Δ^14^C vertical profile there [38]. Assuming this single data point does represent the deep South Atlantic water, this failure might be caused by model deficiencies such as the coarse resolution and eddy-parametrization.

The old deep water Δ^14^C at the LGM has been suggested to be caused by several mechanisms besides a slower overturning circulation. One mechanism is the reduced diapycnal mixing in the abyssal AABW due to the lifting of the interface between the AABW and NADW at the LGM [26,39]. Another mechanism is the expanded sea ice. The Δ^14^C age in the deep ocean represents the combination of surface boundary condition (reservoir age) and the overturning circulation (true water ventilation age). Reservoir age depends mainly on the air–sea gas exchange efficiency and the surface ocean mixing with subsurface ocean (e.g. [40,41]). During glacial periods, the increased reservoir age over the SO due to the extended sea ice coverage (marked in figures 3*g,h* and 6*d,e*) and the mixing with deep water could lead to the older Δ^14^C age than the true water age for the AABW, as suggested previously in observations [4] and models [38,42–44], including CTRACE. In CTRACE, the true ventilation age was represented by the ideal age, which started from 0 on the surface and increased with time along circulation. The ideal age exhibited an overall younger age at the LGM than at the PD (figure 6*g–i*), because of the increased transport of the AABW (see later discussion on figure 9). Rather, this older Δ^14^C age resembled more closely the model v-age (figure 6*d–f*), which was defined the same as the ideal age except for taking into account the insulation effect of sea ice coverage analogues to that in Δ^14^C [45], suggesting that the older radiocarbon age at the LGM was contributed by the sea ice expansion, instead of circulation slowdown in CTRACE [27]. This has implications for the understanding of the carbon cycle, because air–sea exchange of CO_2_ is affected by the sea ice similar to Δ^14^C and v-age.

### (b) Mechanism for glacial water mass formation

One early leading hypothesis for the change of glacial water masses was the shift of westerly wind over the Southern Ocean (SO) [46–48]. Later modelling studies, however, found that the shift of SO wind was too weak (usually less than 10%) to cause a large change in water mass as observed at the LGM [38,49–53]. This weak LGM wind change was consistent with reconstructions [54]. Instead, the enhanced buoyancy flux over SO has emerged as a robust mechanism for the expansion of AABW at LGM [49]. At the LGM, the approximately 100 ppm lowering of atmospheric CO_2_ concentration led to a global cooling and, in turn, a northward expansion of sea ice coverage of up to 1000 km over the SO in reconstructions [55,56] and models (e.g. CTRACE marked in figures 4*g,h* and 6*d,e*). The expansion of sea ice enhanced the brine injection in austral winter, generating the extremely saline and cold AABW at the LGM, as discussed in figure 5. The AABW was so dominant that it became the sole major deepwater mass. The enhanced deep stratification lifted the NADW to the depth of approximately 2000 m, creating the so-called glacial North Atlantic Intermediate Water. This led to a water mass scenario dramatically different from the two-end-member scenario at the PD (figure 3–6). This mechanism of sea ice induced SO buoyancy forcing on glacial water mass transformation was first proposed based on an LGM simulation in a coupled CGCM [49,50], and has since been confirmed robustly across models [38,57–61].

From the coupled ocean–atmosphere perspective, ultimately, this stronger SO buoyancy forcing was caused by the greater sea ice expansion in response to global cooling in the SO than in the Arctic at long time scales, likely because of the thinner sea ice in the SO, which is produced by deep mixing and upwelling there. This also implies a long response time scale for establishing the glacial water mass, because of the slow evolution of SO circulation associated with the AABW and abyssal flows, typically over a thousand years. This long response time may be one reason why the shallow NADW/AMOC has been difficult to simulate in CGCMs. So far, most CGCMs have failed to simulate a shallower NADW/AMOC at the LGM [62–66]. This model failure has been suggested to be caused by the model deficiencies in the formation and export of sea ice and/or brine injection in the SO [61,65,66], the parametrization of topographic-induced mixing [67] and their too strong wind responses over the North Atlantic [64], and it could also be caused by their insufficient length of simulation (mostly less than 1000 years), such that the model AABW had not reached a quasi-equilibrium response [65].

Finally, water masses have also undergone substantial millennial variability during the deglaciation associated with meltwater flux. Model simulations for the millennial variability of water masses have been compared with proxy records. A model-data comparison showed that millennial variability of ε_Nd_ reflected mostly the mixing between the AABW and NADW end-member water masses, instead of biological activities; and furthermore, mid-ocean water mass mixing depended on the changes in not only mixing processes, but also the end members [22]. The CTRACE model-data comparison of the δ^18^O of benthic foraminiferal calcite (δ^18^O*_c_*) between the North and South Atlantic suggested that during HS1, the NADW nearly collapsed, while the AABW weakened only mildly, with the phase difference likely contributed by an abyssal warming, instead of relative water mass contribution between the AABW and NADW [23]. Some other models have also compared their millennial variability with proxy data (e.g. [12,68]) in idealized water hosing experiments (e.g. [69–72]).

### (c) Relevance to the present and future

Based on the study of glacial water mass, it can be speculated that buoyancy forcing can be a major forcing for large changes in deep water masses in the future climate change. It has been well recognized that the SO westerly wind forcing and the associated northward Ekman transport are critical for driving the pole-to-pole AMOC circulation [46,73,74]. This critical role of SO wind on the climatological AMOC, however, does not guarantee that wind change is the dominant forcing for water mass changes and the AMOC during the glacial–interglacial period and for future climate changes [75]. As discussed before, the relative change of westerly wind over the SO is small during the deglacial–interglacial cycle or in future global climate change (e.g. [50–53]), due to the lack of dramatic change of meridional temperature gradient on the global scale. This is in contrast to the local surface buoyancy forcing over the limited deep convection regions [49], which can change dramatically in response to global climate change. In addition, the wind stress impact on the overturning via surface Ekman transport and, in turn, the tilting of isopycnal slopes may be compensated by the increased eddy-driven circulation (see [76] for a review). Finally, the change of surface wind over SO tends also to be decoupled from the wind stress in winter because of the greater sea ice coverage. As such, some models simulate a strengthening and poleward-migration of westerly wind in the atmosphere, consistent with the indications from dust records, but equatorward shift of westerly wind stress over the ocean, consistent with proxy indications of the shift of oceanic fronts [77]. This decoupling effect, however, may become less important in the future global warming scenario, because of the reduced coverage of sea ice. Finally, tidal mixing may be an important mechanism for abyssal circulation and overturning circulation [78]. At the LGM, the large lowing of sea level (approx. 120 m) can trap tidal mixing energy to around abyssal topography, instead of the continental shelf, and therefore enhance abyssal mixing and the global overturning circulation [67,70]. If the sea level rises substantially in the future warming scenario, the opposite may occur.

## 3. AMOC strength since the last glaciation

### (a) Proxy constraint on AMOC strength

The detection of the change of AMOC strength is more challenging than in its geometry. Although δ^13^C, ε_ND_ and Δ^14^C are effective in constraining water mass and AMOC geometry, their effectiveness for constraining the AMOC strength has remained debatable. In an inverse modelling study, Legrand & Wunsch [79] suggested that carbon isotopes were not effective in constraining the AMOC strength, albeit effective in constraining the AMOC depth. Some later modelling studies, nevertheless, suggested that the LGM carbon isotopes indicated a shallower and weaker AMOC than PD [30,37]. However, in these models, a shallower AMOC was always accompanied with a weaker transport, leaving it unclear if the model-data fit was the fit in depth or strength.

Most recent studies suggested that none of δ^13^C, ε_ND_ and Δ^14^C was reliable for detecting the change of AMOC strength. In CTRACE, the AMOC was shallower, but slightly stronger, at the LGM than PD (17.6 Sv. versus 15.6 Sv.). Yet, the model fit with LGM δ^13^C observations was, at least, comparable with the shallower and weaker AMOC in Menviel *et al*. [37] and Muglia *et al*. [30]. To separate the constraints on depth from strength, a weaker and shallower AMOC state was selected in CTRACE. At the 17.5 ka (HS1), forced by meltwater flux, the AMOC transport (approx. 8 Sv.) was 50% weaker than that of PD and the LGM, yet with a shallower depth comparable with that at LGM. The model fit with observational δ^13^C and ε_Nd_ at the HS1 were, however, comparable with those at the LGM. This suggested that these two tracers only constrain the depth, instead of the strength, of the AMOC [27]. The Δ^14^C age was not a good proxy of the AMOC strength either. In addition to the sea ice impact discussed earlier, the deepwater age is a measure of the transport weighted from all water sources and is therefore determined by not only the strength of the AMOC. These results were further confirmed by Muglia & Schmittner [80] with a comprehensive series of experiments that consist of various combinations of depth and strength of the AMOC in the UVic model.

**Figure 7.**
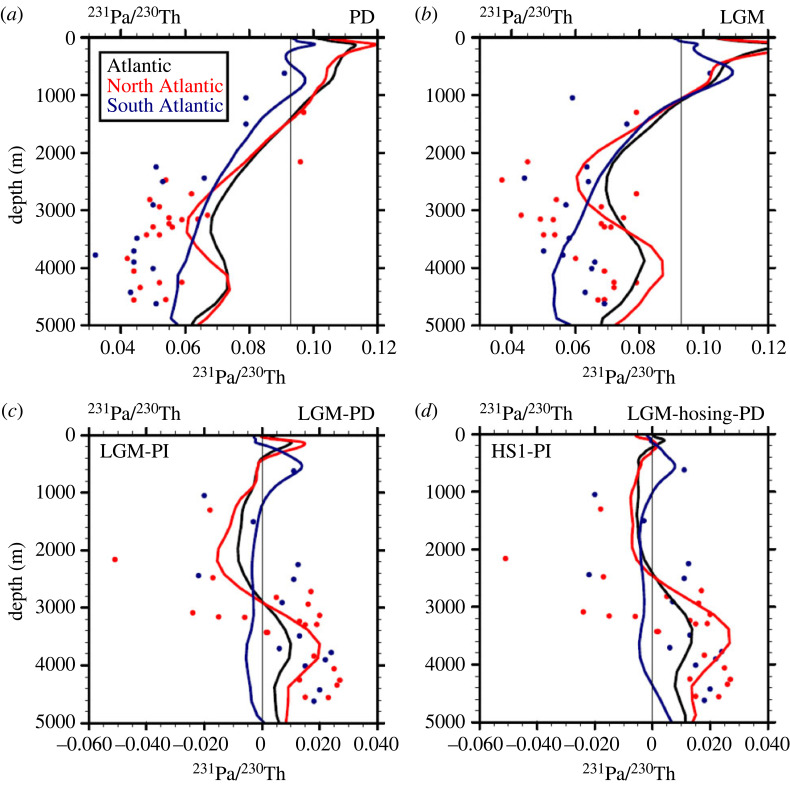
Atlantic ^231^Pa/^230^Th profiles: Atlantic average (black); North Atlantic average (10° N–45° N) (red); South Atlantic average (10° S–34° S) (blue): (*a*) PD, (*b*) LGM, (*c*) difference between LGM and PD; (*d*) difference between HS1 and PD. The ^231^Pa/^230^Th observations are plotted in dots, with observations from the North Atlantic in red and observations from the South Atlantic in blue. Maximum AMOC transport (depth) is 16 Sv. (3000 m) for PD, 18 Sv. (2500 m) at LGM and 10 Sv. (2300 m) for HS1. See Gu *et al*. [27] for more details (adapted from [27]).

**Figure 8.**
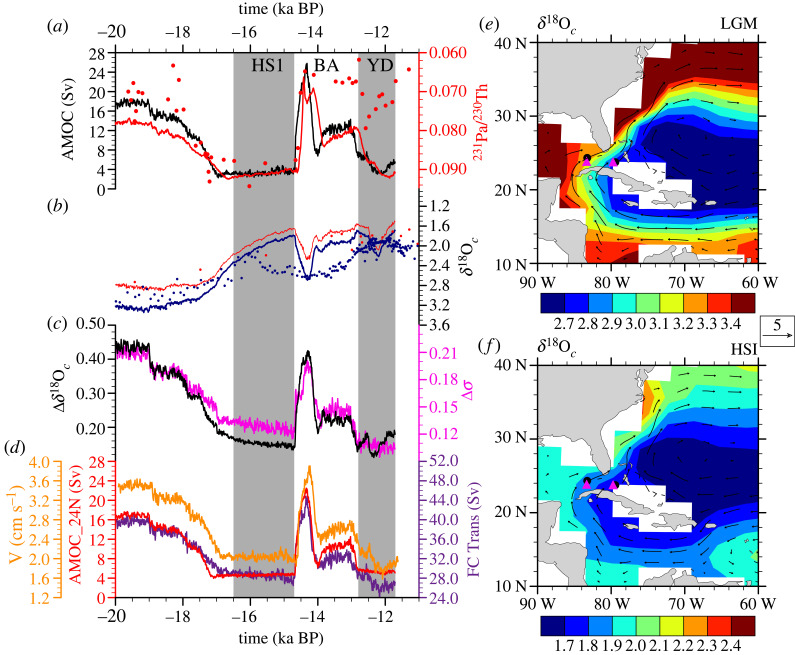
Time evolutions in the Florida Straits and the δ^18^O*_c_* distribution in C-iTRACE. (*a*) AMOC strength (black) and observational (red dot) and model (red curve) ^231^ Pa/^230^Th from site OCE326-GGC5 (33°42′ N, 57°35′ W, 4.55 km). (*b*) δ^18^O*_c_* evolution from two cores located on either side of the Florida Current (blue for the west site; red for the east site). Dots are observations, and curves are model results. The location of the two observational sites in Lynch-Stieglitz *et al.* [83] is shown as black circles, and the location of the model grids for these two observational sites are shown as magenta triangles in (*f*). (*c*) δ^18^O*_c_* contrast (black) and density contrast (magenta) between the two Florida Straits sites in C-iTRACE. (*d*) The average meridional velocity at 530 m in the Florida Straits (orange), the Florida Current transport (purple) and the maximum of the Atlantic meridional overturning stream function at 24.5° N (red). (*e*) The Sverdrup transport in the Florida Straits in C-iTRACE. (*f*) δ^18^O*_c_* distribution at 530 m during LGM in C-iTRACE, with velocity overlaid as vectors. Adapted from Gu *et al*. [22,84].

Sediment ${}_{}^{231}\text{Pa/}{}_{}^{\text{230}}\text{Th}$ has been suggested as a proxy sensitive to the strength of AMOC export of the NADW [6,81,82]. A lower value of ^231^Pa/^230^Th (relative to its production rate of 0.093) in the deep North Atlantic is likely caused by a stronger AMOC export of the NADW, because of a longer residence time of ${}_{}^{231}\text{Pa}$ than ${}_{}^{230}\text{Th}$. The iconic ^231^Pa/^230^Th in the Bermuda Rise has been used as a proxy for the AMOC strength during the last glacial cycle [6] (figures 1*e* and 2*g*). The ^231^Pa/^230^Th sensitivity to AMOC strength was confirmed in CTRACE [24,27]. As shown in figure 7*a,b*, the lower ^231^Pa/^230^Th tongue in the deep Atlantic in both the PD and LGM corresponded to an active AMOC export in both observations and model. The difference between the LGM and PD (figure 7*c*) showed an upward shift of the low ^231^Pa/^230^Th in the North Atlantic in both the model and observations, corresponding to the shallowing AMOC as discussed on δ^13^C and ε_Nd_. The ^231^Pa/^230^Th sensitivity to AMOC strength was also seen in comparing the differences between HS1-PD and LGM-PD in the model: the weaker model AMOC in HS1 (8 Sv) than LGM (16 Sv) could indeed be seen in the smaller depletion of ^231^Pa/^230^Th difference in the former than latter (solid lines in figure 7*d* versus *c*). The ^231^Pa/^230^Th variability during the last deglaciation was also consistent with the AMOC variability in CTRACE (figure 2*g*, also later in figure 8*a*). Note, however, that ^231^Pa/^230^Th is sensitive to both the strength and geometry of the AMOC. Therefore, a more accurate representation of AMOC transport would depend on the ^231^Pa/^230^Th record, not only on a single site of, say, McManus *et al*. [6], but also on other sites [81,85,86].

**Figure 9.**
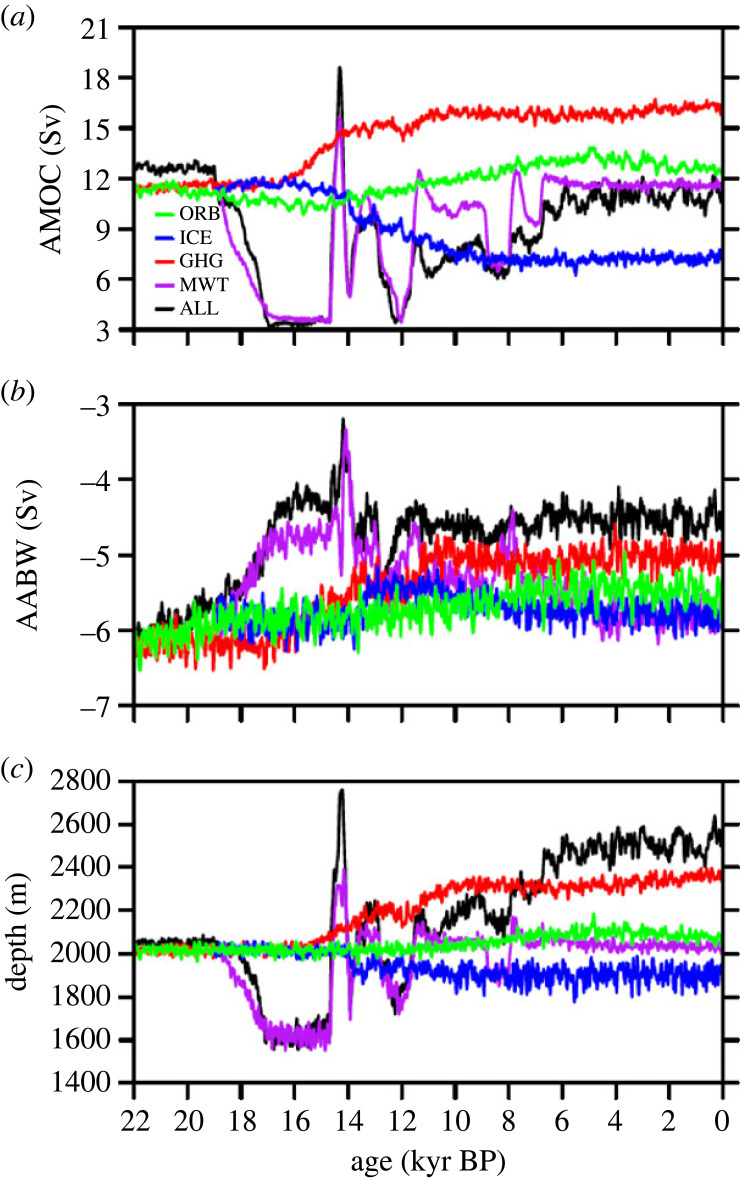
TRACE deglacial evolutions of (*a*) AMOC intensity (units: Sv = 10^6^ m^3^ s^−1^), defined as the maximum of the positive (northern sinking) overturning streamfunction below 500 m in the North Atlantic, (*b*) AABW intensity (units: Sv), defined as the minimum of the negative streamfunction over the Atlantic basin, and (*c*) AMOC depth (units: m), defined as the depth of the mid-depth zero streamfunction averaged in the Atlantic basin in ALL (black), GHG (red), ICE (blue), ORB (green) and MWT (purple) runs. Adapted from Zhu C *et al*. [75].

The δ^18^O in foraminifera (δ^18^O*_c_*) can be used to infer AMOC strength because δ^18^O*_c_* is a useful proxy for density. The zonal contrast of the δ^18^O*_c_* can therefore be used as a proxy of density contrast and, in turn, the circulation transport through the thermal wind relation [87]. Lynch-Stieglitz *et al.* [7] first attempted a reconstruction of the transport of the Florida Current using δ^18^O*_c_* (figure 1*g*, [3]), which was supposed to reflect the strength of the upper branch of the AMOC return flow. The CTRACE model confirmed that the δ^18^O*_c_* difference across the Florida Strait was indeed a good proxy for the variation of Florida Current transport and, in turn, the AMOC transport during the last deglaciation (figure 8, [84]). It has been further proposed that the δ^18^O*_c_* contrast zonally across the South Atlantic basin could be used to reconstruct the AMOC meridional transport over the Atlantic basin [25,87,88]. CTRACE model simulations, however, showed somewhat inconsistent results, given the data available (not shown). The reason δ^18^O*_c_* was successful in the reconstruction of the strength of the Florida Current, but not in the basin-wide AMOC in the South Atlantic, was that in the warm upper ocean of Florida Current, density was determined mainly by the temperature of calcification, which dominated the variability of δ^18^O*_c_* as in the relation of δ^18^O*_c_* = δ^18^O*_w_* − 0.27 + 0.0011*t*^2^ − 0.245*t* + 3.58, where δ^18^O*_w_* is sea water δ^18^O and *t* is temperature in ^o^C [89]. In the South Atlantic, deep ocean salinity variability becomes important and the complex salinity structure associated with the AAIW made it difficult to reconstruct the cross-basin density contrast accurately using δ^18^O*_c_* alone. The situation seemed similar in the reconstruction of the transport of the Antarctic Circumpolar Current (ACC), which has the additional complexity of different fractionations of δ^18^O*_c_* in sea ice than in water [90,91]. Nevertheless, with additional information of temperature or salinity (or δ^18^O*_w_*), especially at the level of AAIW, it might be possible to reconstruct the transports more credibly [91].

### (b) Mechanism for glacial AMOC transport

As inferred from the proxy records of ^231^Pa/^230^Th and δ^18^O*_c_* gradient, the strength of the AMOC has changed dramatically during the last glacial–interglacial cycle (figure 1*f,g*). The most prominent feature is the millennial events of collapsed AMOC during the Heinrich Stadial events (HS), which could be forced by meltwater fluxes in CGCMs as studied previously, for example, in TRACE (figure 2). More interesting in the proxies, however, is the roughly comparable AMOC strength between the LGM and PD, in contrast to the dramatic difference in water masses as discussed in §2. Indeed, the ^231^Pa/^230^Th records ranged from somewhat weaker than [6] and indistinguishable from [81], to somewhat stronger than [85,86] the PD, depending on the location of the proxy. Combining ^231^Pa/^230^Th reconstructions with a two-dimensional tracer model suggested that the AMOC during the LGM could be at least as strong as that of the PD [92]. Some other studies also suggested little difference in the AMOC transport [31,93], although only δ^13^C and nutrient data were assimilated in these works. Published CGCM simulations of the LGM AMOC showed agreement on neither the geometry nor the strength of the AMOC from the earlier PMIP2 and PMIP3 to the most recent PMIP4 (Paleoclimate Modeling Intercomparison Project) [62–64,66]. This inconsistency of model AMOC strength has been attributed to various reasons, including the differences in diapycnal mixing schemes [67], Antarctic sea ice formation [65], model-specific bias [94], ice sheet impact on Northern Atlantic wind stress [64], and initial conditions and insufficient equilibrium time [65,94]. These seemingly inconsistent results in reconstructions and models may also imply that the AMOC is unstable and thus very sensitive to the boundary conditions and other modelling and forcing details.

One alternative interpretation was that AMOC intensity was not much different between the LGM and PD, neither too much stronger nor too much weaker, at least relative to the periods of the HS events. If this interpretation was correct, it raised a dynamical question: How could the AMOC be of comparable intensity at the LGM and PD, given the significant difference in the surface climate forcing associated with the change of the greenhouse gases (GHGs) and the ice sheet (figure 2*b,c*)? In the ocean, besides the dramatic change of the deep water mass as discussed earlier, there was also likely significant change in the deep convection in the North Atlantic, with the deep convection region shifted from the GIN Sea at the PD to the subpolar North Atlantic at the LGM. This shift was caused by the expansion of winter sea ice to over the entire GIN Sea at the LGM [55], suppressing deep convection there in models [75,95–97]. It is possible that the AMOCs were at different climate states at the LGM and PD and therefore have different responses. This argument, however, does not provide any physical insight unless the physical mechanisms at different states are identified. Current modelling studies seemed to be consistent with one interpretation: the comparable AMOC transport was caused by the opposing effects between the GHGs and the ice sheet forcing on the AMOC in the coupled ocean–atmosphere system [27,75]. This interpretation was motivated by the study of the evolution of AMOC transports (along with the AABW abyssal cell transport and the depth between the two cells) in a set of sensitivity experiments under individual forcing factors applied in the TRACE simulation (figure 9). While the full TRACE experiment (ALL in figure 9) was forced by the realistic GHGs, ice sheet, orbital forcing and meltwater forcing combined (figure 2*a–c*), the GHG, ICE, ORB and MWT experiments are each forced by one individual forcing factor of the GHGs, ice sheet, orbital forcing and meltwater forcing, respectively. From the LGM to PD, the AMOC transport increased substantially with the rising GHGs (from approx. 12 Sv to almost 16.5 Sv, red in figure 9*a*), but decreased substantially with the retreating ice sheet (from approx. 12 Sv to almost 7.5 Sv, blue in figure 9*a*), with little response to orbital and meltwater forcing. As such, the AMOC transport under the full forcing (ALL, black), which is almost a linear superposition of the AMOC transports under individual forcing, becomes comparable at the LGM and PD due to the cancellation between the opposing responses to the rising GHG and retreating ice sheet. Furthermore, these opposing responses to deglacial changes of GHGs and ice sheet were consistent with those in two independent models [94,96]. This compensation response posed several questions. First, why did the AMOC intensify with the rising GHGs, but weaken with the retreating ice sheet? Second, why did the AMOC intensify with the rising GHGs during the deglaciation, but weaken in response to increased anthropogenic GHGs?

The increased AMOC transport with GHGs was opposite to the robust AMOC weakening response to the rising CO_2_ in the future. This difference occurred because the CO_2_ rose slowly during the deglaciation (figure 2*b*), forcing a long-term quasi-equilibrium response that was the opposite to the initial short-term (decadal to centennial) response [95,98]. This initial weakening followed by later recovery of the AMOC with rising CO_2_ was also evident in earlier studies in a CGCM [99] and Earth System Models of Intermediate Complexity (EMICs) [100] and, most recently, in the LongRunMIP simulations [101,102]. With the rapid CO_2_ rise, the increased surface heat flux warmed SST in the North Atlantic, which reduced the surface buoyancy flux loss over the deep convection region and therefore weakened the AMOC [103]. With a slow CO_2_ rise, however, the long adjustment time of the AMOC was enhanced by two mechanisms. First, the sea ice cover was reduced over the GIN Sea (Greenland, Iceland, and Norwegian Seas), which increased the heat loss to the atmosphere and enhanced the deep convection in the North Atlantic and, in turn, the AMOC [95,104]. Second, the warming over the SO reduced the sea ice coverage and the associated brine injection, which weakened the AABW transport (red in figure 9*b*) and the deep water stratification in the North Atlantic [50,59,98], increasing the depth of the pycnocline/AMOC and eventually the AMOC transport [73]. The rate of the AMOC recovery, however, seemed to be state-dependent. The first enhancing mechanism was most effective at the LGM when the sea ice covered broadly over the North Atlantic and GIN Sea. The second enhancing mechanism was most effective under a slow CO_2_ forcing because it became effective only after the slow adjustment of the AABW. Since both recovery mechanisms depended on the sea ice and the associated positive feedback, the recovery was much stronger at the LGM than at PD, because of the greater sea ice cover at the LGM [95].

In contrast to the AMOC response to the rising CO_2_, the retreating Laurentide ice sheet forced the North Atlantic storm track migrating northward, which enhanced the surface wind stress over subpolar North Atlantic and then drove a sea ice expansion there, reducing heat loss to the atmosphere and eventually the AMOC intensity, as shown robustly across models [96,105–107]. From the global circulation perspective, a further analysis of TRACE simulations showed that, the increased export of the NADW with rising CO_2_ is upwelled diabetically mainly with a shallowing overturning in the Indo-Pacific basin via the enhanced inter-basin exchange, while the reduced AMOC with retreating ice sheet was accompanied by reduced adiabatic pole-to-pole circulation associated with the reduced westerly wind stress over the SO [75].

It was also interesting to note in TRACE that the AMOC transport response to the total forcing was almost a linear superimposition of the responses to all individual forcing factors (figure 9*a*), similar to the global temperature. A similar linear superposition was also found in the response of the AABW bottom cell transport, which weakened from the LGM to PD (figure 9*b*). By contrast, the response of the AMOC depth was far from a linear superposition, where the AMOC depth deepened significantly under the full forcing, in spite of the cancellation of the deepening response forced by GHGs and the shallowing response forced by ice sheet (figure 9*c*). Given the strong nonlinear nature of the AMOC, this linearity of its transport response is not obvious.The robustness and mechanism of the response linearity remains to be further explored.

### (c) Relevance to the present and future

Current theories have highlighted the SO westerly wind and diapycnal mixing as two major drivers of the pole-to-pole AMOC transport [46,73,74]. These two forcing factors, however, did not seem to be able to explain the AMOC responses in the current deglacial CGCM experiments. The lack of large change in the SO wind and abyssal mixing between the LGM and PD could be speculated to offer an explanation of the comparable AMOC transports at the LGM and PD in the ALL forcing experiment, but cannot explain the large change of AMOC strengths in the single forcing experiments GHGs and ICE. By contrast, the buoyancy forcing seemed to provide a much more robust driver of the AMOC transport change across the experiments, reminiscent of the response of deepwater mass discussed earlier. This robust role of the buoyancy forcing can be reconciled with the current theories as follows. First, in the current theory, buoyancy forcing can also change AMOC transport indirectly by changing deep stratification [73,74]. Second, current theories assume a single pole-to-pole cell atop a stagnant abyssal layer and can be modified by the addition of an abyssal cell associated with the AABW. Finally, it is much easier for global climate forcing to change local buoyancy forcing over deep convection region than to change the basin-wide wind stress and deep mixing. One caveat in the above argument is that interactive tidal mixing schemes have not been implemented in these CGCMs, so its role remains to be explored.

The AMOC response during deglaciation is helpful for our understanding of the AMOC response to both CO_2_ and the ice sheet in the future. During deglaciation, the slow change of atmospheric CO_2_ was almost in phase with the retreating ice sheet, leading to the cancellation of these two opposite forcing effects. In the future, atmospheric CO_2_ is likely to increase much faster than the decline of ice sheet over Greenland and Antarctica. Therefore, one may expect a fast AMOC response to CO_2_ forcing alone, followed by the response to the slow ice sheet change thousands of years later. The response to future ice sheet could also differ from that during the deglaciation, leading to different atmospheric and, in turn, AMOC responses. Furthermore, the recovery response to long-term CO_2_ forcing is likely to be even weaker than for the PD, because the warming will reduce the sea ice coverage further. Finally, the different constraints on the AMOC transport and water masses suggest that AMOC strength and depth are controlled by different forcing factors and therefore may not increase, or decrease, together.

## 4. Summary and perspective

### (a) Summary

Current simulations of the AMOC during the last deglaciation have led to the following conclusions.

First, different proxies constrain different aspects of the AMOC. The distribution of water mass and AMOC geometry can be constrained well by δ^13^C, and ε_Nd_, while the AMOC strength is better constrained by ^231^Pa/^230^Th and δ^18^O gradient. While Δ^14^C is the tracer most relevant to water ages, the inferred age can be substantially older than the true water age from the surface due to the shielding by sea ice coverage, especially from the SO during glacial period. It remains to be studied if, and how, Δ^14^C can be used to infer true circulation changes.

Second, deglacial deep water mass can be changed most effectively by the change of surface buoyancy forcing over the localized region of deepwater formation, especially over the SO. The SO wind forcing, although important for forcing the climatological mean AMOC, may not change substantially in response to certain climate change, and therefore may not be the dominant driver of AMOC change.

Third, AMOC response to external climate forcing can differ dramatically between initial short time scale and later long-term responses. It can also be opposite to different forcing during the deglaciation and possibility in the future. However, due to the different time scales of external forcing variation, paleo experience may not be applied directly to future projections, especially in the near future of centennial time scale.

### (b) Perspective

With the improvement of both data and model, it is the time to combine data and model optimally using data assimilation (DA) to reconstruct past ocean state. Proxy observations will continue to accumulate. Ocean models will be improved not only in their physical processes, but also in the incorporation of key paleo geotracers such that data can be directly assimilated into models. Computational power has increased to the stage that ensemble simulations of the continuous deep ocean evolution of approximately 10 000 years are possible in non-eddy resolving OGCMs. This makes it possible to perform ensemble DA [108], as in present-day weather and climate DA (e.g. [109]). Ensemble DA has been used in the last decade for paleoclimate DA, for the last millennium [110], the LGM [111,112] and the deglacial evolution since LGM [9]. However, these DA studies have focused on the surface temperature. There are past attempts to reconstruct ocean circulation and water mass at the LGM using other DA approaches, on selective types of proxy data [31,93]. Now, it is possible to perform ensemble DA for the entire deglacial evolution of the Atlantic water masses and circulation with more comprehensive proxy data.

Compared with the AMOC reconstruction in the present-day DA [113,114], the reconstruction of paleo AMOC, and ocean state in general, are much more challenging. A key challenge for paleo DA, in general, is the estimation of the uncertainties in both the data and model. Paleoceanography records for the deglacial period are very sparse and tend to be located in certain regions, notably continental margins. The uncertainty in data must include not only the measurement error, but also representative error, which could be particularly large for paleo proxies. For models, the forcing for the ocean model should ideally be generated using coupled models, or CGCMs, with synchronous coupling, such that the ocean evolution and, in turn, the surface climate fields are not distorted artificially. It is also important to understand the sensitivity of different ocean state variables to different proxies such that the available data can be used to reconstruct those oceanic variables most credibly. Finally, model uncertainty should be evaluated using multiple models for the ensemble such that the analysis is not biased by a specific model [112]. Unlike surface temperature, deep ocean has memory of thousands of years, and therefore on-line assimilation should provide the best analysis product, in principle. The on-line approach, however, is practically challenging if multi-models are used for the ensemble. As the first step, therefore, it is more practical to use multi-model ensemble with off-line DA methods [110,112,115] such that each simulation can be performed in individual model independently. A coordinated international project of multiple modelling groups should be formed for this effort [116].

## Data Availability

This article has no additional data.
